# Optimization of Sonication Parameters to Produce a Cashew Apple Bagasse Puree Rich in Superoxide Dismutase

**DOI:** 10.3390/foods11172694

**Published:** 2022-09-03

**Authors:** Thatyane Fonteles, Ana Karoline Leite, Thaiz Miguel, Fabiano Fernandes, Sergimar Pinheiro, Emílio Miguel, Sueli Rodrigues

**Affiliations:** 1 Department of Food Engineering, Federal University of Ceara, Fortaleza 60440900, Brazil; 2 Department of Chemical Engineering, Federal University of Ceara, Fortaleza 60455760, Brazil; 3 Department of Metallurgical and Materials Engineering, Federal University of Ceara, Fortaleza 60440554, Brazil

**Keywords:** *Anacardium occidentale*, antioxidative enzymes, toxicity, *Artemia salina*, ultrasound

## Abstract

The effects of ultrasound processing parameters on the extraction of antioxidative enzymes and a toxicity assessment of cashew apple bagasse puree were investigated. Ultrasound directly affects the formation of reactive oxygen species such as H_2_O_2_, and consequently, superoxide dismutase, catalase, and ascorbate peroxidase activities. S.O.D. activity increased up to 280% after U.S. processing at 75 W/cm^2^, 1:3 bagasse: water ratio, and 10 min compared to non-processed bagasse. Therefore, the effect of ultrasound in delaying browning could be correlated to the enhanced antioxidant enzyme activity and decrease in peroxidase activity. At center point conditions (226 W/cm^2^, 1:3 bagasse: water ratio; 6 min), a decrease of 20% and 50% on POD and PPO activities was observed, respectively. No significant acute toxicity or protective effect was observed in unprocessed and sonicated cashew apple bagasse. Although cashew bagasse processed at 75 W/cm^2^ prevented nauplii death after 24 h of exposure, this data cannot assure the protective effect once the number of dead nauplii on 100 μg/mL was similar. However, these data indicate a possible protective effect, especially in higher cashew bagasse concentrations. The results suggest that sonicated cashew apple bagasse puree, a coproduct obtained from a traditional valued fruit in Brazil, may be used as a source of antioxidative enzymes, which further has great importance in therapeutics.

## 1. Introduction

Reactive oxygen species (R.O.S.) formation is inherent to aerobic organisms occurring at a controlled rate in healthy cells. The oxidative stress arises when an asymmetry between the formation of R.O.S. and their neutralization through enzymatic or non-enzymatic control systems is observed, resulting in subsequent alteration of proteins, membrane lipids, and nucleic acids. Oxidative stress is a cellular phenomenon associated with several pathologies, including atherosclerosis, cardio and neurodegenerative disorders, and carcinogenesis [1]. Eating fruits is important in well-balanced nutrition as a source of natural bioactive compounds [2]. Thus, it has been suggested that antioxidant supplementation could have health-promoting effects [3,4,5,6,7].

As most plants such as roots [8], fruits [9,10,11], and cereals [12,13] contain a substantial amount of antioxidant enzymes, the extraction and identification of antioxidant molecules from plants become an up-and-coming food research area [13]. However, commercial preparations of antioxidative enzymes such as superoxide dismutase (S.O.D.) or catalase (C.A.T.) are expensive.

Superoxide dismutase is an antioxidant defense of aerobic organisms that catalyzes superoxide anion reduction into peroxides, which are scavenged by C.A.T., ascorbate peroxidase (A.P.X.), and other common enzymes [1,14]. In plants, S.O.D. isoforms are located in the mitochondria (Mn-SOD), cytosol (Cu/Zn-SOD), and chloroplasts (Fe-SOD) with different physiological functions.

S.O.D.s extracted from plants have been evaluated in human studies as interventions in different pathologies such as diseases related to oxidative stress and aging [1]. Hua et al. [15] have extracted S.O.D. from black ginger and observed a protective effect on the liver of type 2 diabetic rats. However, in some cases, plant-derived S.O.D. shows low oral bioavailability [9]. S.O.D. can be obtained by protein extraction from plants. S.O.D. extraction methods should not involve solvents, which require additional purification steps resulting in S.O.D. denaturation. There is, thus, a need for new, simple, fast, and low-cost methods for S.O.D. obtainment [16].

The use of non-thermal extraction technologies can improve the antioxidant potential of plant-based foods due to the higher concentration of bioavailable, bioactive compounds after processing. Ultrasound (U.S.) processing has been used as a non-thermal, cheap, eco-friendly, and easy-to-use technology to extract nutraceuticals from plants, including phenolic compounds, vitamins, and proteins [17]. U.S. is also known as an elicitor with several effects on cell functions, including the formation of R.O.S. and the activity of antioxidative enzymes [18,19]. U.S. effects come from mechanical and chemical events [20]. Mechanical effects are observed after the growth and implosion of cavitation bubbles, resulting in localized simultaneous high temperatures and pressures. Chemical effects are caused by the formation of R.O.S., such as H_2_O_2_, with possible side effects for consumers of U.S. processed foods [21,22].

Extracting S.O.D. in clean extracts will make possible the application of the extracts in human food and animal feed. In addition, it is worth noting that the extracts obtained by U.S. contain other beneficial bioactive compounds naturally present in plants, such as polyphenols, which have antioxidant properties [23].

Some safety aspects of U.S. food processing still need deeper exploitation as R.O.S. generation is involved in certain human diseases. Therefore, considering the promising effects of U.S. food processing and the scarcity of toxicological trials regarding non-thermal food processing, U.S. processing was optimized for cashew apple bagasse (C.A.B.) to release antioxidative enzymes, enhancing C.A.B. bioactive content. Furthermore, the toxicity assessment and the protective effect of the sonicated bagasse were evaluated using *Artemia salina* as a biological model.

## 2. Materials and Methods

### 2.1. Cashew Apple Bagasse

The cashew apple is an abundant fruit cultivated in India, Vietnam, Brazil, and other African and Asia countries. Cashew apple bagasse (C.A.B.) is the coproduct from cashew apple peduncle processing. It represents a low-cost source of bioactive compounds such as phenolics and vitamin C. Mature red cashew apple peduncles collected directly from the producer (Fortaleza, Ceara, Brazil) were sanitized by immersion in 2 g/L of sodium hypochlorite solution for 1 min and after the juice was extracted by pressing. The final solid residue (bagasse) was sealed and stored at −20 °C.

### 2.2. Sonication

C.A.B. sonication was conducted in U.S. equipment with 500 W of total power input and probe macropoint (1.3 cm ø) (Unique**^®^** DES500, São Paulo, Brazil). The processing was performed in 600 mL glass beaker immersing the macroprobe 1.5 cm in the liquid without temperature control. A 2^3^ central composite design with 17 runs (3 center points) was conducted to evaluate C.A.B. sonication effects. The independent parameters evaluated were power intensity, the bagasse: water ratio, and the processing time (Table 1).

Statistica software (T.I.B.C.O. Software Inc. (2020). Data Science Workbench, version 14. http://tibco.com (accessed on 8 October 2021)) was used to build the plots from the response surface analysis. A control sample for each bagasse: water ratio was used. The samples were submitted to water extraction without sonication at 25 °C for 10 min.

### 2.3. Enzyme Extraction

For S.O.D., C.A.T., and A.P.X. activities determinations, enzyme extraction was conducted according to Wissemann and Lee [24]. Sonicated C.A.B. (2 g) was homogenized in 15 mL 0.1 M K-phosphate buffer, pH 7.0 containing 0.1 mM EDTA, and centrifuged (4000× *g* for 10 min at 4 °C, model Sigma 6-16KS, Sigma, Germany). For peroxidase (P.O.D.) and polyphenol oxidase (P.P.O.) determinations, 10 g of sonicated C.A.B. were mixed with 10 mL of 0.05 M K-phosphate buffer (pH 7.0) containing 1% (*w*/*v*) polyvinylpyrrolidone (PVP) and centrifuged (10,000× *g* for 20 min at 4 °C, model Sigma 6-16KS, Sigma Zentrifugen, Germany). All reagents used for enzyme extraction were from Sigma-Aldrich (St Louis, MO, USA).

### 2.4. Protein Determination

The supernatant fraction was used as a crude extract for the protein content determination according to Bradford [25].

### 2.5. Enzyme Assays

The reaction for S.O.D. determination consisted of 50 µL enzyme extract diluted in 1000 µL 50 mM K-phosphate buffer (pH 7.8) with 0.1 μM EDTA and 19.5 mM methionine. In light absence, 0.15 mL 75μM N.B.T. and 300 µL 10 mM riboflavin were added. The reaction was exposed to fluorescent light (20 W) for 15 min. The absorbance readings were made at 560 nm where one unit of S.O.D. activity (U.A.) was defined as the amount of enzyme to cause a 50% reduction of N.B.T. The results were expressed as U.A./mg of protein [26].

For C.A.T. measuring, the reaction started by adding 60 µL H_2_O_2_ and 10 µL enzyme extract to 1430 µL 0.1 M K-phosphate buffer plus EDTA 0.1 mM pH 7.0 at 30 °C. The decrease in H_2_O_2_ was read at 240 nm (molar extinction coefficient 36 M/cm). One unit of C.A.T. activity (U.A.) was defined as the amount of enzyme required to decompose H_2_O_2_ (μmol H_2_O_2/_min), and the results were expressed as U.A./mg of protein [27].

A.P.X. was measured by adding 50 µL enzyme extract, 50 µL H_2_O_2_, and 50 µL ascorbic acid to 1350 µL of 0.05 M K-phosphate buffer with EDTA 0.05 mM pH 6.0 at 30 °C. The results were expressed in μmol H_2_O_2_/mg^−1^ P/min using the molar extinction coefficient for ascorbate (2.8 mM/cm) according to Nakano and Asada [28]

P.P.O. was measured according to Wissemann and Lee [24]. The reaction mixture contained 300 µL enzyme extract and 1850 µL 0.1 M K-phosphate buffer pH 6.0 containing 0.1 M catechol and 0.1 M KCl at 30 °C. After 30 min, the reaction was interrupted with 800 µL HClO_4_ 2 N. One unit of enzyme activity (1 U.A.) was defined as the amount of enzyme that causes a change of 0.001 in the absorbance measured at 395 nm per min.

P.O.D. was monitored at 470 nm according to Matsuno and Uritani [29]. The P.O.D. activity was measured as follows: 2750 µL 0.1 M Na-phosphate-citrate buffer pH 5.0 containing 1% (*v*/*v*) guaiacol and 250 µL of 3% H_2_O were added to 1500 µL enzyme extract, and the assay was incubated at 30 °C. After 5 min, the reaction was interrupted with 1000 µL of 30% (*w*/*v*) NaHSO_4_. Therefore, one unit of enzyme activity (1 UEA) was defined as the amount of enzyme that causes a change of 0.001 in the absorbance per min. All reagents used for enzyme activity determination were from Sigma-Aldrich (St Louis, MO, USA).

### 2.6. H_2_O_2_ Content

H_2_O_2_ content was measured according to Mapelli et al. [30]. Sonicated bagasse (0.2 g) was homogenized with 5000 µL 0.1% T.C.A. and then centrifuged at 12,000× *g* for 15 min. A total of 500 µL supernatant was mixed with 500 µL 10 mM K-phosphate buffer pH 7 and 1000 µL 1 M K.I. The absorbance was measured at 390 nm. The H_2_O_2_ content was expressed in μmol H_2_O_2_/g. All reagents used for the determination of H_2_O_2_ content were from Sigma-Aldrich (St Louis, MO, USA).

### 2.7. Expression of Results

The results were expressed as residual activities determined according to Equation (1)

(1) $$\mathrm{Residual}\;\mathrm{activity}\;\left(\%\right)=100\cdot \frac{A_{s}}{A_{0}}$$

The sub-indices 0 and s mean the control sample (non-processed) and the sonicated one, respectively. Three control experiments at different bagasse: water ratio were also evaluated, and the residual activity was calculated for each bagasse: water ratio.

### 2.8. Color

The color of the C.A.B. puree after sonication was determined using a Minolta CR300 colorimeter (Tokyo, Japan). The reflectance instruments determined lightness (L), redness (a), yellowness (b), C (Chroma), and h° (hue angle). The total color difference was calculated according to Equation (2). The reference value for ΔE was the non-sonicated bagasse.

(2) $$\Delta E=\sqrt{{\left(\Delta L\right)}^{2}+{\left(\Delta a\right)}^{2}+{\left(\Delta b\right)}^{2}}$$

### 2.9. Toxicity against Artemia salina

*Artemia salina* cysts (0.1%) were incubated in artificial seawater for 48 h at 24 °C under 16 h-light, 8 h-dark, and continuous aeration. Positive phototaxis was used to distinguish the hatched nauplii from the non-hatched cysts. Then, the hatched nauplii were transferred to 24-well culture plates containing artificial seawater [31].

#### 2.9.1. Toxicity Assays

The toxicity assays were conducted with 10, 100, and 1000 μg/mL of sonicated C.A.B. administered in nauplii Instar II within 24 and 48 h at 25 °C changing 8-h dark/16-h light on 24-well plates. Each replicate contained 10 newly hatched nauplii. A negative control experiment was conducted with artificial seawater, and the positive control experiment was exposed to 0.5 M potassium dichromate (K_2_Cr_2_O_7_) (Sigma-Aldrich, St Louis, MO, USA). The dead larvae were counted using a stereomicroscope Zeiss Stem 508 (Zeiss, Dresden, Germany). The test is considered valid when the survival rate in the control group was superior to 90% [32].

#### 2.9.2. Protective Effect of Sonicated Cashew Apple Bagasse Puree against H_2_O_2_

Nauplii Instar II were exposed to their H_2_O_2_ LC_50_ [21]. In 24-well microplates containing artificial seawater, Instar II nauplii at 10, 100, and 1000 μg/mL were exposed to 127.45 mM (LC_50_) H_2_O_2_. After 2 h of H_2_O_2_ nauplii interaction, sonicated C.A.B. was added to wells. Counting and evaluating the animal morphological, changes were conducted after 24 and 48 h. In the negative control experiment, nauplii were exposed to artificial seawater and in the positive control to 127.45 mM H_2_O_2_.

### 2.10. Morphological Assays

#### 2.10.1. Light Microscopy

The nauplii collected after 24 h for toxicity and protective assay, washed in seawater, were prepared on a glass slide. The possible morphological changes were observed at a light microscope Primo Star-Zeiss equipped with Zen Light software (Zeiss, Dresden, Germany).

#### 2.10.2. Scanning Electron Microscopy (S.E.M.)

*A. salina* nauplii instar II were collected as described above and fixed in 2.5% glutaraldehyde, 4.0% formaldehyde in 0.1 mol/L cacodylate buffer, pH 7.2, at 25 °C during 24 h. Then, the samples were washed in 0.1 mol/L sodium cacodylate buffer three times for 45 min each and post-fixed for 1 h at 25 °C with 1% osmium tetroxide in 0.1 mol/L cacodylate buffer. Finally, the samples were acetone dehydrated until the critical point (Q150T ES) for 45 min each step. Dried samples were placed in stubs sputtered with a 20-nm gold. Observation and documentation were performed in a scanning electron microscope (Quanta FEG 450 FEI, Waltham, MA, USA) [21]. All reagents used for S.E.M. experiments were from Sigma-Aldrich (St Louis, MO, USA).

### 2.11. Statistical Analysis

The results were expressed as mean ± standard deviation. F-test and ANOVA analyses were used as the significant parameter for the fitted models. The significant differences among means were determined using Tukey’s test (*p* < 0.05). Statistical analysis of the experimental data was performed using the software Statistica (T.I.B.C.O. Software Inc. (2020, Munich, Germany). Data Science Workbench, version 14. http://tibco.com (accessed on 8 October 2021)).

## 3. Results

### 3.1. Activity of Antioxidative Enzymes

**Figure 1:**
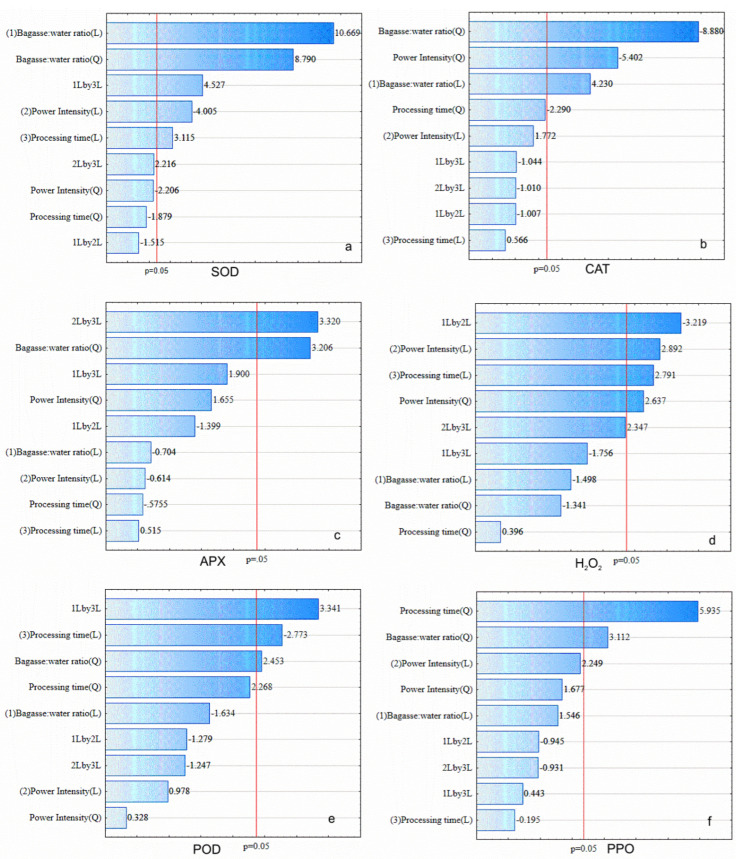
Pareto charts for the effect of sonication on S.O.D. (superoxide dismutase) (**a**), C.A.T. (catalase) (**b**), A.P.X. (ascorbate peroxidase) (**c**), H_2_O_2_ (hydrogen peroxide content) (**d**), P.O.D. (peroxidase) (**e**), P.P.O. (polyphenol oxidase), and (**f**) activities of cashew apple bagasse.

The response surface methodology was employed to study the effects of the independent variables power intensity, bagasse: water ratio, and processing time (Figure 1a). The sonication of C.A.B. affected the activity of S.O.D., C.A.T., and A.P.X. antioxidative enzymes. S.O.D. activity increased up to 280% compared to non-processed bagasse (Table 2). Furthermore, the proportion of water added to bagasse before sonication (bagasse: water ratio presented a higher positive significance on S.O.D. residual activity (*p* < 0.05) i.e., more water added to bagasse increases S.O.D. activity. In addition, the interactive effect of bagasse: water ratio and processing time were positive. Conversely, the linear effect of U.S. intensity within the experimental range evaluated showed that higher power intensity led to S.O.D. reduction since this effect was negative (Figure 1a).

**Figure 2:**
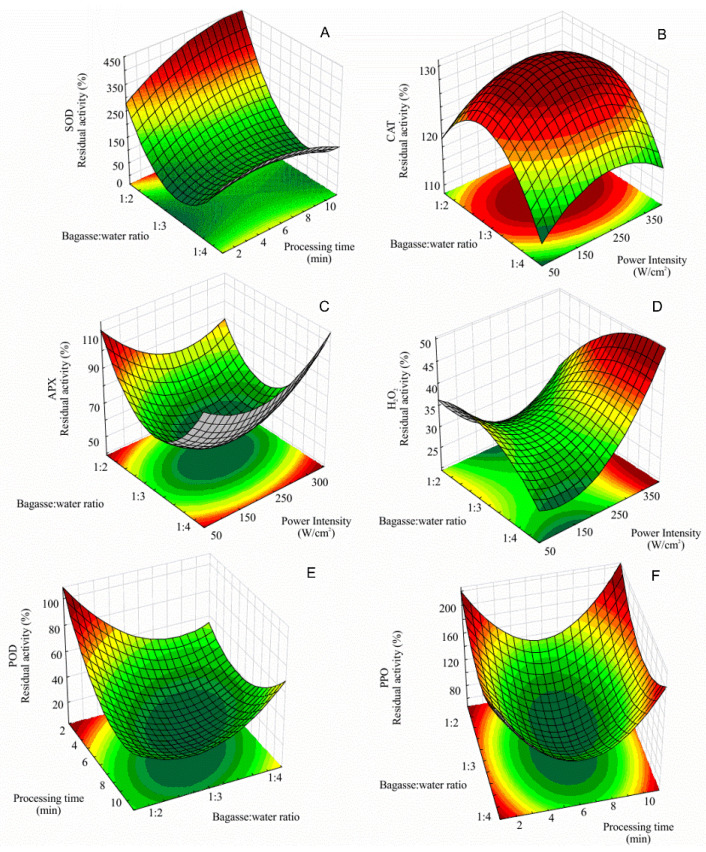
Response surface for the effect of sonication on SOD (superoxide dismutase) (**A**), CAT (catalase) (**B**), APX (ascorbate peroxidase) (**C**), H_2_O_2_ content (**D**), POD (peroxidase) (**E**), PPO (polyphenol oxidase) and (**F**), activities of cashew apple bagasse.

The fitted model for residual S.O.D. activity as a function of bagasse: water ratio, U.S. power intensity, and the processing time is expressed in Table S1. An inflection point on the surface shows a local minimum for processing time and a local maximum for bagasse: water ratio (Figure 2a). When the processing time was ≈6 min, S.O.D. residual activity increased for bagasse: water ratio higher and lower than 1:3. A similar result was found for U.S. processing of bayberry juice by Cao et al. [33] with an increment of S.O.D. activity processed at 90–450 W/cm^2^ for 4 min was observed.

S.O.D. is a defense mechanism against the toxicity of the superoxide anion and its intermediates [34]. The mechanical stress induced by U.S. cavitation and microstreaming can stimulate the activity of antioxidative enzymes, such as S.O.D., which is stimuli-responsive [35,36]. Otherwise, the increased activity can be attributed to the extraction effect caused by U.S. sonication. In the cell, S.O.D. isoenzymes are compartmentalized. For instance, manganese S.O.D. (MnSOD) is in the mitochondria; copper and zinc S.O.D. (Cu-ZnSOD) is in the cytoplasm and the nucleus [6]. The cavitation caused by U.S. processing of liquid media can generate high localized temperatures and pressures. As a result, cell walls can be disrupted, and the release of the bioactive extractable compounds can be facilitated [37,38].

The same result was observed for C.A.T. residual activity, which was 19% higher than the non-processed sample. In addition, the quadratic effects of bagasse: water ratio and power intensity presented a negative effect on C.A.T. residual activity (*p* < 0.05) (Figure 1b). The model for the residual C.A.T. activity as a function of bagasse: water ratio, U.S. power intensity, and the processing time is expressed by Table S1. Figure 2b depicts the surface response plot built from Table S1.

The response surface plot shows a well-defined area for maximum C.A.T. residual activity. The critical point of the U.S. processing conditions for maximal C.A.T. residual activity is where the first derivate of Table S1 equals zero. Thus, the processing is maximized at a power intensity of 236 W/cm^2^, 1:3 bagasse: water ratio for 6 min.

The impact of U.S. on the A.P.X. activity of C.A.B. is summarized in Table 2. The effects of the independent variables on A.P.X. residual activity are shown in Figure 1c. The bagasse: water ratio (quadratic) and the interaction between power intensity and processing time positively affected A.P.X. residual activity (*p* < 0.05). Hence, a simultaneous increase in U.S. power intensity and processing time increased A.P.X. activity. Conversely, a marked decrease (≈50%) of A.P.X. activity was observed at the center point (226 W/cm^2^, 1:3 and 6 min), the same processing parameters that favored C.A.T. activity (Figure 2c). The U.S. extraction of scavenging antioxidative enzymes from C.A.B. may be an alternative resource to defend against oxidative stress.

The results of the experimental design for H_2_O_2_ residual concentrations after sonication are presented in Table 2. U.S. processing caused a significant increase in H_2_O_2_ content of sonicated C.A.B., reaching the maximum concentration on assay 4 (373 W/cm^2^, 1:2 bagasse: water ratio and 10 min).

Figure 1d shows the effects of the evaluated variables on H_2_O_2_ concentration after sonication. The results showed that all U.S. processing variables, at distinct levels, contributed to the final concentration of H_2_O_2_ of sonicated C.A.B. However, U.S. intensity exerts a higher positive effect.

This tendency was also observed by Kentish and Ashokkumar [39] who reported that the generation of free radicals by U.S. was higher when the higher temperature inside the cavitation bubble was achieved. The bubble temperature can be increased by increasing the U.S. power and the external pressure or decreasing the external temperature. The fitted model for residual H_2_O_2_ concentration after sonication is expressed by Table S1. The levels of H_2_O_2_ are critical once it mediates the response involved in the oxidative stress [6]. R.O.S. formation is considered a challenge for food bioactive compounds’ preservation, such as polyphenols [40]. At the higher U.S. power intensity, the U.S. effect on H_2_O_2_ production increased with exposure time (Table 2). Herein, S.O.D. and C.A.T. increased activities after U.S. processing of C.A.B. may indicate its effective scavenging mechanism to remove H_2_O_2_ as a protective role of these enzymes against oxidative stress induced by sonication.

C.A.T. residual activity was most important in removing H_2_O_2_ of sonicated C.A.B. because its action was more significant than the A.P.X. It appears to be associated more clearly with the content of H_2_O_2_.

The results for P.O.D. residual activity of C.A.B. after sonication were presented in Table 2. The effects of the independent variables on the P.O.D. residual activity are shown in Figure 2e. Results for P.O.D. activity showed that the effect of processing time (linear) was significant (*p* < 0.05) on the P.O.D. activity reduction. Conversely, the effect of power intensity (linear and quadratic) was not significant. The effect of U.S. power intensity showed that, within the experimental range evaluated, higher power intensities combined with low processing times did not affect P.O.D. activity reduction since this effect was insignificant. An increase in P.O.D. activity was found for simultaneous high bagasse: water ratio and processing times (Figure 1e).

The critical point of Table S1 calculated the minimal residual activity (supplementary material). The model predicted 80% of P.O.D. inactivation with the following processing conditions: power intensity 150 W/cm^2^, 1:2 of bagasse: water ratio for 6.9 min (Figure 2e).

Browning reactions have been associated with a consequence of P.P.O. and P.O.D. action on polyphenols [41]. Figure 1f shows the single and interactive effects of the independent variables on P.P.O. residual activity. The Pareto chart shows that increasing processing time and power intensity results in higher P.P.O. activities.

The surface response plot for P.P.O. residual activity is presented in Figure 2f. The minimal activity of P.P.O. was determined by the critical point of Table S1. The P.P.O. activity was minimized at power intensity 168 W/cm^2^, 1:2 of bagasse: water ratio for 5.97 min reaching 74% of residual activity.

The behavior of P.P.O. observed here is, at first, due to U.S. processing that causes cell disruption of the C.A.B. tissue. Thus, intracellular P.P.O. can be released, increasing the enzyme activity. However, at the experimental design center point (226 W/cm^2^, bagasse: water ratio 1:3 and 6 min), the enzyme denaturation overcomes the enzyme release (Figure 2f).

The U.S. processing affects enzyme activity through single or both mechanical and chemical effects. As a result, R.O.S. formation through water sonolysis, thermal effect, cavitation, and micro-streaming could affect the integrity of the protein chain [42]. In addition, sonolysis causes water decomposition to H^+^ and OH^−^, which would bind to the protein aminoacidic chain, affecting enzymatic activity [33].

**Figure 3:**
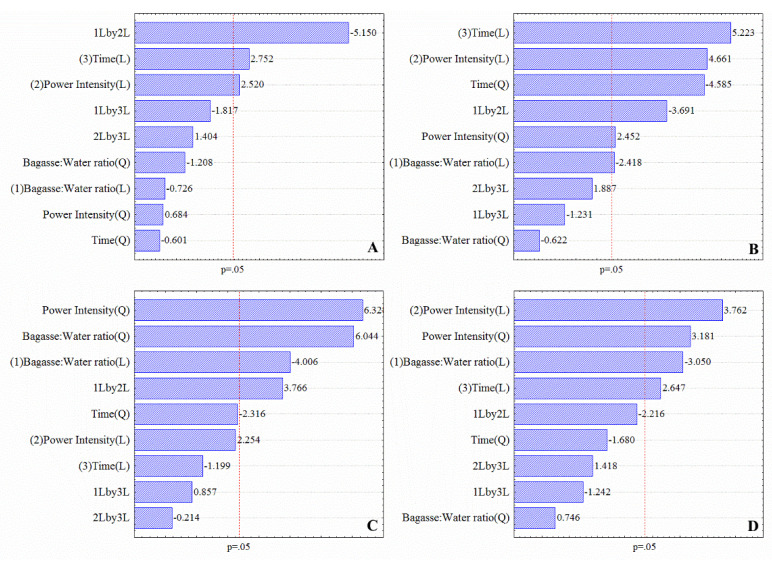
Pareto charts for the effect of sonication on luminosity (L) (**A**); hue angle (h°) (**B**); Chroma (ΔC) (**C**); and total color difference (ΔE) (**D**) of sonicated cashew apple bagasse (C.A.B.).

Regarding the effects of sonication on C.A.B. color, U.S. power intensity (linear), processing time (linear), and the interaction of power intensity and bagasse: water ratio presented significant positive effects on L* values. Thus, in the experimental domain evaluated herein, sonication improves the luminosity of the sonicated cashew apple puree (Table S2, Figure 3).

**Figure 4:**
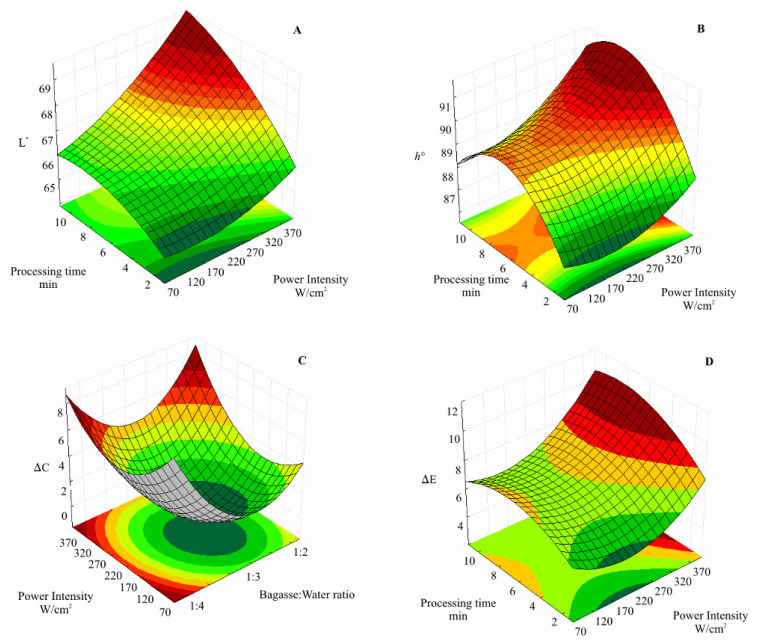
Response surface for the effect of sonication on luminosity (L*) (**A**); hue angle (h°) (**B**); Chroma (ΔC) (**C**); and total color difference (ΔE) and (**D**); of sonicated cashew apple bagasse (C.A.B.).

Despite some changes, the hue angle observed for sonicated C.A.B. was an average of 65°, representing the characteristic color of C.A.B. This value is between 0° (red) and 90° (yellow). The effects of power intensity (linear and quadratic) and linear effects of bagasse: water ratio and processing time showed positive effects on ΔE at a 95% of confidence level (Figure 4). A ΔE > 2 indicates visually perceptible differences [43]. Thus, the sonication resulted in perceptible color changes in C.A.B. puree. Despite this result, there was no evidence of browning since h° of sonicated samples was close to the characteristic color of the cashew apple.

During the U.S. processing, cell membranes are disrupted, conferring a better homogenization and intensifying the yellow color of C.A.B. According to Xu et al. [8], there is a positive correlation between R.O.S. accumulation and enzymatic browning. Therefore, in this study, the effect of U.S. in delaying browning could be correlated to the enhanced antioxidant enzyme activity and a decrease in guaiacol peroxidase activity.

### 3.2. Toxicity and Protective Effect against Artemia salina

**Figure 5:**
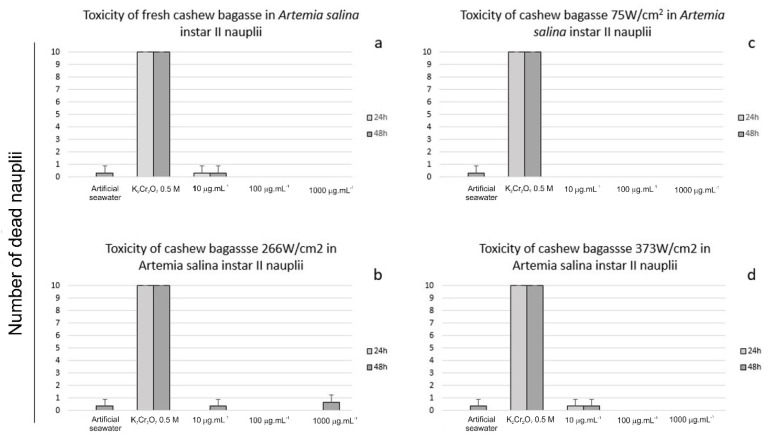
Acute toxicity of sonicated cashew apple bagasse (10, 100, and 1000μg/mL) in Artemia salina instar II nauplii at 24 and 48 h of exposure. No significant acute toxicity was noted in fresh (**a**), 75 W/cm^2^ (**b**), 266 W/cm^2^ (**c**), or 373 W/cm^2^ (**d**), sonicated cashew apple bagasse at 24 or 48 h of exposure. In addition, negative control (artificial seawater) did not cause significant death of nauplii. Conversely, positive control (K_2_Cr_2_O_7_ 0.5 M) was lethal for 100% of individuals, including the 24 h of exposure.

No significant acute toxicity was noted in unprocessed (Figure 5a), 75 W/cm^2^ (Figure 5b), 266 W/cm^2^ (Figure 5c), or 373 W/cm^2^ (Figure 5d) sonicated cashew bagasse at 24 or 48 h of exposure. The number of dead nauplii was higher at 48 h in all treatments.

**Figure 6:**
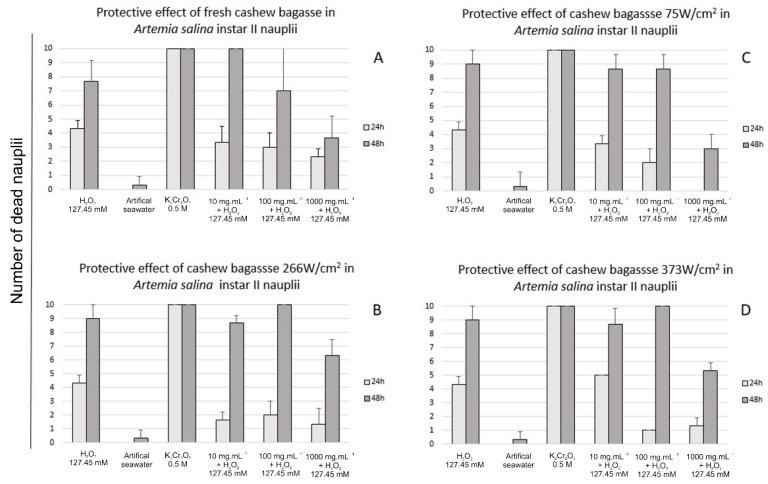
Protective effect of sonicated cashew apple bagasse (10, 100, and 1000 μg/mL) in *Artemia salina* instar II nauplii at 24 and 48 h of exposure. No significant protective effect was noted in fresh (**A**), 75 W/cm^2^ (**B**), 266 W/cm^2^ (**C**), or 373 W/cm^2^ (**D**) sonicated cashew bagasse at 24 or 48 h of exposure. Negative control (artificial seawater) did not cause significant death of nauplii. Conversely, positive control (K_2_Cr_2_O_7_ 0.5 M) was lethal for 100% of individuals, including 24 h of exposure. H_2_O_2_ at LC_50_ (127.45 mM) cause death of nearly 50% of individuals at 24 h exposure and death of nearly all nauplii at 48 h of exposure.

No significant protective effect was observed for unprocessed (Figure 6A), 75 W/cm^2^ (Figure 6B), 226 W/cm^2^ (Figure 6C), or 373 W/cm^2^ (Figure 6D) sonicated cashew bagasse at 24 or 48 h of exposure. However, cashew bagasse processed at 75 W/cm^2^ (1000μg/mL) prevented nauplii death after 24 h of exposure (Figure 6A). This data cannot assure the protective effect once the number of dead nauplii on 100 μg/mL (Figure 6B) was similar. However, these data indicate a possible protective effect, especially in higher cashew bagasse concentrations. In general, the protective effect is dose-dependent, as Miguel et al. [21] described. Unlike C.A.B., other vegetable drinks such as blueberry juice [44] and fruit wines [45] also promoted a protective effect against oxidative stress. The number of dead nauplii was higher at 48 h at all treatments. According to Miguel et al. [21], 127.45 mM H_2_O_2_ is the lethal concentration for a 24 h experiment. H_2_O_2_ at LC_50_ (127.45 mM) causes the death of nearly 50% of individuals at 24 h exposure. After 24 h of exposure to H_2_O_2_ (127.45 mM) almost all nauplii were dead.

### 3.3. Morphological Evaluation

**Figure 7:**
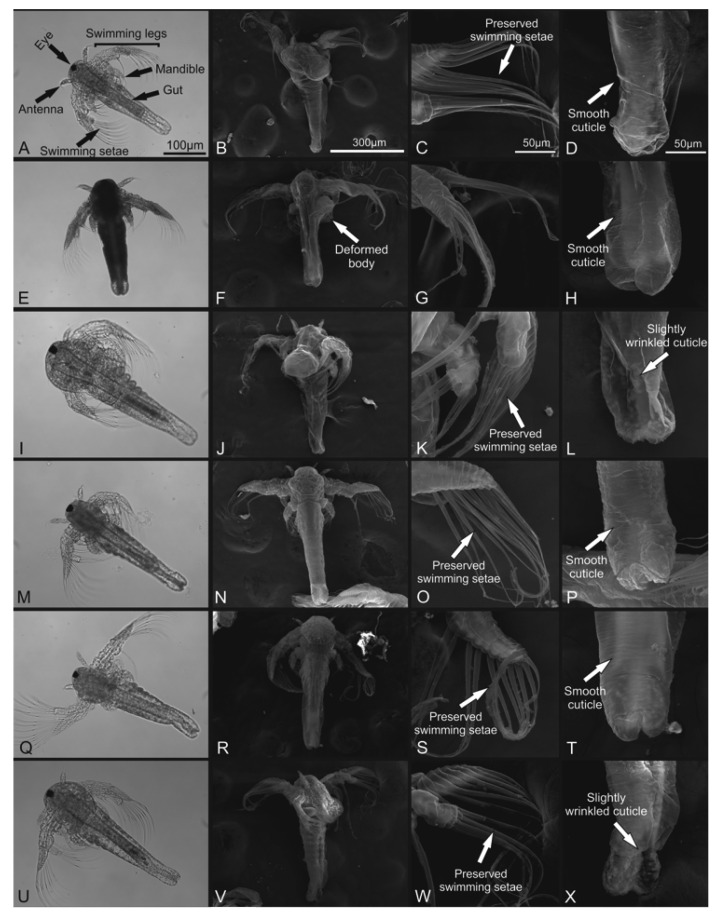
Optical and scanning electron microscopy of *A. salina* nauplii within 24 h exposure on acute toxicity test. (**A**–**D**) Negative control (artificial seawater); (**E**–**H**) positive control (K_2_Cr_2_O_7_ 0.5 M); (**I**–**L**) fresh cashew bagasse 1000 μg/mL; (**M**–**P**) 75 W/cm^2^ cashew bagasse 1000 μg/mL; (**Q**–**T**) 226 W/cm^2^ cashew bagasse 1000 μg/mL; (**U**–**X**) 373 W/cm^2^ cashew bagasse 1000 μg/mL. Note severe damage on nauplii exposed to K_2_Cr_2_O_7_. Bars: (**A**,**E**,**I**,**M**,**Q**,**U**)—500 μm; (**B**–**D**,**F**–**H**,**J**–**L**,**N**–**P**,**R**–**T**,**V**–**X**)—50 μm.

In the toxicity test, a negative control group (seawater) exhibited typical morphology with preserved structures such as antenna and swimming legs (Figure 7A–D). K_2_Cr_2_O_7_ 0.5 M (positive control group) resulted in deformations in the animal’s body and structural loss such as antenna, eye, and appendages (Figure 7E–H). The exposition of *A. salina* nauplii to cashew bagasse did not promote significant morphological changes, including the higher concentration (100 μg/mL) in all treatments (Figure 7I–X).

**Figure 8:**
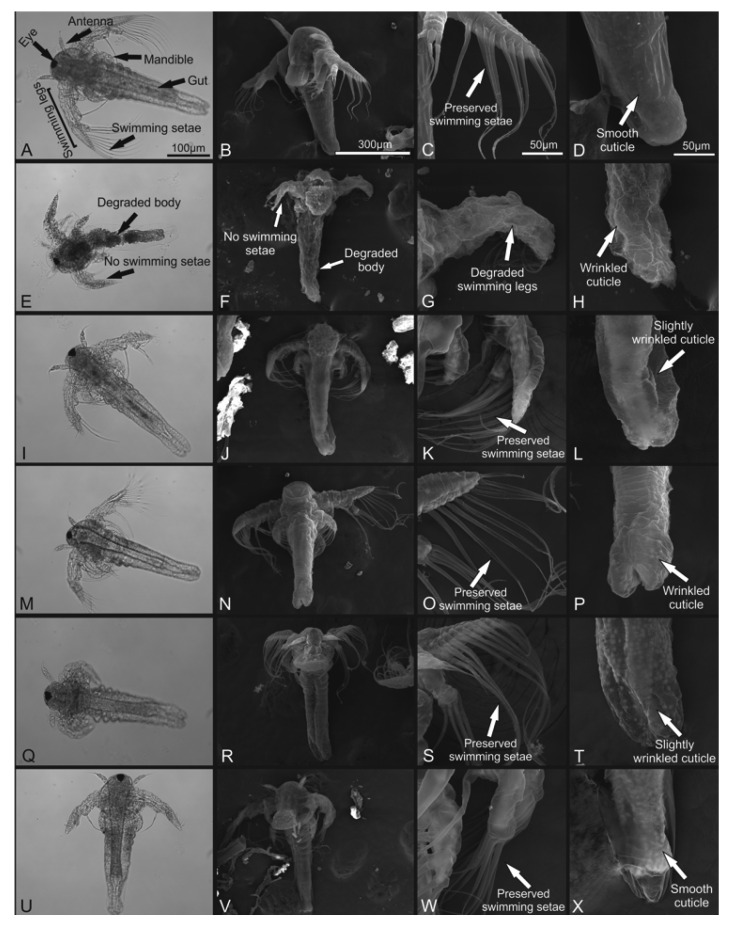
Optical and scanning electron microscopy of *A. salina* nauplii within 24 h exposure on protective effect test. (**A**–**D**) Negative control (artificial seawater); (**E**–**H**) positive control (H_2_O_2_ 127.45 mM); (**I**–**L**) fresh cashew apple bagasse (CAB) 1000 μg/mL; (**M**–**P**) 75 W/cm^2^ CAB 1000 μg/mL; (**Q**–**T**) 226 W/cm^2^ sonicated CAB 1000 μg/mL; (**U**–**X**) 373 W/cm^2^ CAB 1000 μg/mL. Note severe damage on nauplii exposed to hydrogen peroxide presenting degraded body, no swimming legs, and wrinkled cuticle. Bars: (**A**,**E**,**I**,**M**,**Q**,**U**)—500 μm; (**B**–**D**,**F**–**H**,**J**–**L**,**N**–**P**,**R**–**T**,**V**–**X**)—50 μm.

In the protective effect evaluation, the negative control group exhibited typical morphology as previously described (Figure 8A–D). H_2_O_2_ caused morphological changes in *A. salina*, with the loss of the body’s structural integrity (Figure 8E–H), loss of swimming setae and swimming legs, and cuticle wrinkle. The exposure of individuals to cashew bagasse after contact with H_2_O_2_ did not prevent deaths. However, the structural changes in these individuals were milder than in those who did not receive this treatment (Figure 8I–X). Only small morphological changes as wrinkled and slightly wrinkled cuticles were noted (Figure 8L,P,T).

Antioxidative enzymes act by breaking down the toxic superoxide radicals into oxygen and hydrogen peroxide and the increased activity of antioxidative enzymes after C.A.B. sonication could contribute to the observed result [46].

Studies on the morphology of *A. salina* nauplii exposed to a protective effect against oxidative stress are scarce. Most of the studies only deal with the survival and death of individuals [47,48,49]. In these rare works that show a protective effect, the morphology of individuals is preserved compared to those exposed to the stressor [21]. Even though it does not prevent deaths, morphology preservation was observed in nauplii exposed to H_2_O_2_. This fact raises the question of whether increasing the dose has a protective effect on individuals.

## 4. Conclusions

The results collectively indicate that the physical and chemical stimuli from sonication induced the increase in H_2_O_2_ concentration. The activities of S.O.D., C.A.T., and A.P.X. were responsible for neutralizing the toxic effect of H_2_O_2_, converting it into water. Sonication also reduced the activities of the deteriorative enzyme P.O.D. with preservation of color. No significant acute toxicity or protective effects were observed for sonicated cashew apple puree. This result suggests that sonicated C.A.B., a coproduct from Brazil’s traditionally valued tropical fruit, may also act as a potential source of antioxidative enzymes, which further has great importance in different applications such as antioxidants for food and the pharmaceutical industry.

## Figures and Tables

**Table 1:** Independent factors and levels.

Independent Factors	Coded Levels		
	−1	0	+1
Power Intensity (W/cm^2^)	75	226	373
Bagasse: water ratio (g/mL)	1:4	1:3	1:2
Processing time (min)	2	6	10

**Table 2:** Experimental design and S.O.D. (superoxide dismutase), C.A.T. (catalase), A.P.X. (ascorbate peroxidase), P.P.O. (polyphenol oxidase), and P.O.D. (peroxidase) activities and H_2_O_2_ content after sonication of cashew apple bagasse puree.

Assay	Power Intensity (W/cm^2^)	Time (min)	Bagasse: Water Ratio	Residual (%)					
				SOD	CAT	APX	H_2_O_2_	PPO	POD
1	75	2	1:2	171.31 ± 0.08	100.61 ± 0.01	100.00 ± 0.10	84.21 ± 0.03	153.20 ± 2.45	77.32 ± 0.00
2	75	10	1:2	83.96 ± 1.67	112.45 ± 0.01	59.46 ± 0.09	175.00 ± 0.03	173.96 ± 2.89	34.97 ± 0.26
3	373	2	1:2	72.99 ± 0.99	95.91 ± 0.06	74.16 ± 0.23	140.78 ± 0.03	217.13 ± 1.86	98.46 ± 0.88
4	373	10	1:2	126.16 ± 0.24	85.67 ± 0.04	93.21 ± 0.45	486.84 ± 0.03	179.02 ± 2.21	48.18 ± 0.29
5	75	2	1:4	249.00 ± 0.76	108.76 ± 0.00	97.55 ± 0.05	248.68 ± 0.03	185.52 ± 5.72	37.05 ± 0.00
6	75	10	1:4	376.28 ± 0.56	110.86 ± 0.08	91.87 ± 0.09	164.47 ± 0.03	179.30 ± 9.31	56.47 ± 0.00
7	373	2	1:4	162.71 ± 0.97	119.22 ± 0.08	52.11 ± 0.08	109.21 ± 0.03	189.97 ± 2.31	49.41 ± 0.49
8	373	10	1:4	300.09 ± 1.67	108.76 ± 0.00	100.00 ± 0.01	161.84 ± 0.03	200 ± 3.20	39.11 ± 0.29
9	226	6	1:2	172.15 ± 0.54	104.56 ± 0.02	100.00 ± 0.32	109.21 ± 0.03	78.19 ± 0.78	27.61 ± 0.26
10	226	6	1:4	345.77 ± 0.57	112.95 ± 0.64	58.33 ± 0.09	98.68 ± 0.03	125.63 ± 4.29	49.41 ± 0.19
11	75	6	1:2	124.37 ± 0.76	87.71 ± 0.00	64.14 ± 0.00	119.73 ± 0.03	74.75 ± 3. 10	22.92 ± 0.39
12	373	6	1:3	44.11± 0.21	98.68 ± 0.00	72.16 ± 0.29	307.89 ± 0.03	97.50 ± 3.13	28.14 ± 0.36
13	226	2	1:3	92.01 ± 0.12	104.16 ± 0.09	53.45 ± 0.00	115.78 ± 0.03	127.05 ± 3.28	45.31 ± 0.54
14	226	10	1:3	85.29 ± 0.53	97.06 ± 0.08	50.00 ± 0.07	156.57 ± 0.03	129.59 ± 4.23	31.07 ± 0.00
15	226	6	1:3	103.36 ± 0.36	85.78 ± 0.01	51.00 ± 0.15	102.63 ± 0.03	83 ± 1.20	23.30 ± 0.19
16	226	6	1:3	105.00 ± 1.04	87.42 ± 0.03	49.77 ± 0.29	105.00 ± 0.03	84.57 ± 0.88	22.92 ± 0.67
17	226	6	1:3	102.07 ± 0.45	85.99 ± 0.05	47.00 ± 0.04	102.00 ± 0.03	85.54 ± 0.99	22.92 ± 0.36

## Data Availability

The data presented in this study are available on request from the corresponding author.

## Supplementary Materials

The following supporting information can be downloaded at: https://www.mdpi.com/article/10.3390/foods11172694/s1, Table S1: Fitted model for dependent variables as a function of bagasse: water ratio, U.S. power intensity, and processing time; Table S2: Effect of ultrasound on the color of cashew apple bagasse puree.
