# Supplementary material for: Optimization of Sonication Parameters to Produce a Cashew Apple Bagasse Puree Rich in Superoxide Dismutase
Source: Foods. 2022 Sep 3;11(17):2694. doi: 10.3390/foods11172694 (PMC9455690; doi:10.3390/foods11172694)
Supplement: Supplementary file 1 [file foods-11-02694-s001.zip › foods-1817289-supplementary.pdf]

Supplementary materials

Table S1. Surface response fitted models.

**Enzyme**	**Model**	$\mathbf{R^2}$	$\mathbf{F_{9,7}}$
SOD	Y=1906.20−9882.23x+12638x2−0.01y−0.001y2+44.09z −1.72z2+0.74xy−68.21xz+0.03yz	0.97	19.89
CAT	Y=73.52+253.80x−364.81x2+0.070y−0.0002y2+1.04z−0 .092z2+0.02xy+0.68xz−0.001yz	0.97	33.92
APX	Y=312.57−1175.60x+1634.48x2−0.50y+0.001y2+3.73z− 0.26z2+0.27xy−14.89xz+0.02yz	0.83	3.94
H_2O_2	Y=-109.16+1147.17x-385.51x2- 1.08y+0.004y2+14.41z+0.79z2-2.14xy-69.71xz+0.082yz	0.93	11.06
POD	Y=144.43−582.98x+960.86x2−0.07y+0.0001y2−2.49z+0 .92z2+0.26xy−25.26xz−0.008yz	0.89	6.41
PPO	Y=347.18−628.51x+756.13x2−0.12y+0.001y2−43.60z+3 .66z2−0.18xy+3.08xz−0.009yz	0.95	15.45

F – Fisher test t 95% of confidence interval

Table S2. Sonication effect on CAB puree color

Assay	L	h°	ΔC	ΔE
1	66.41 ± 0.05	87.77 ± 0.07	2.24 ± 0.00	6.31 ± 0.04
2	67.13 ± 0.00	88.72 ± 0.01	2.26 ± 0.00	7.20 ± 0.03
3	64.40 ± 0.01	87.59 ± 0.02	5.22 ± 0.02	7.08 ± 0.01
4	65.01 ± 0.03	89.09 ± 0.01	5.17 ± 0.09	7.63 ± 0.01
5	63.27 ± 0.00	87.36 ± 0.04	6.21 ± 0.06	7.17 ± 0.00
6	64.64 ± 0.00	88.53 ± 0.11	5.66 ± 0.02	7.56 ± 0.10
7	65.81 ± 0.10	88.79 ± 0.19	6.03 ± 0.00	8.39 ± 0.04
8	71.03 ± 0.03	91.87 ± 0.03	5.15 ± 0.01	12.3 ± 0.12
9	65.27 ± 0.09	89.51 ± 0.00	3.22 ± 0.04	6.70 ± 0.00
10	65.68 ± 0.05	89.67 ± 0.00	3.57 ± 0.00	7.26 ± 0.13
11	64.68 ± 0.01	89.44 ± 0.34	4.28 ± 0.00	7.01 ± 0.05
12	68.60 ± 0.00	91.70 ± 0.01	3.26 ± 0.10	9.89 ± 0.00
13	65.56 ± 0.01	88.07 ± 0.02	0.84 ± 0.03	5.43 ± 0.04
14	66.28 ± 0.02	89.14 ± 0.01	-0.41 ± 0.00	6.68 ± 0.03
15	67.06 ± 0.03	89.28 ± 0.02	0.61 ± 0.00	7.13 ± 0.01
16	67.67 ± 0.01	89.70 ± 0.00	0.62 ± 0.00	8.25 ± 0.01
17	66.59 ± 0.01	89.22 ± 0.00	0.61 ± 0.07	6.72 ± 0.02
**Control**				
	**L**	**h°**	**Chroma**	
1:2	63.10 ± 0.01	84.99 ± 0.09	43.28 ± 0.07	--
1:3	61.90 ± 0.00	84.65 ± 0.05	42.09 ± 0.03	--
1:4	60.79 ± 0.00	83.32 ± 0.00	40.27 ± 0.02	--
